# Supplementary material for: Cumulative Weighing of Time in Intertemporal Tradeoffs
Source: J Exp Psychol Gen. 2016 Sep;145(9):1177–205. doi: 10.1037/xge0000198 (PMC4998108; doi:10.1037/xge0000198)
Supplement: Supplementary file 1 [file Scholten_Individuals.zip › plots/e30_p311_eg2_priors_and_posteriors.pdf]

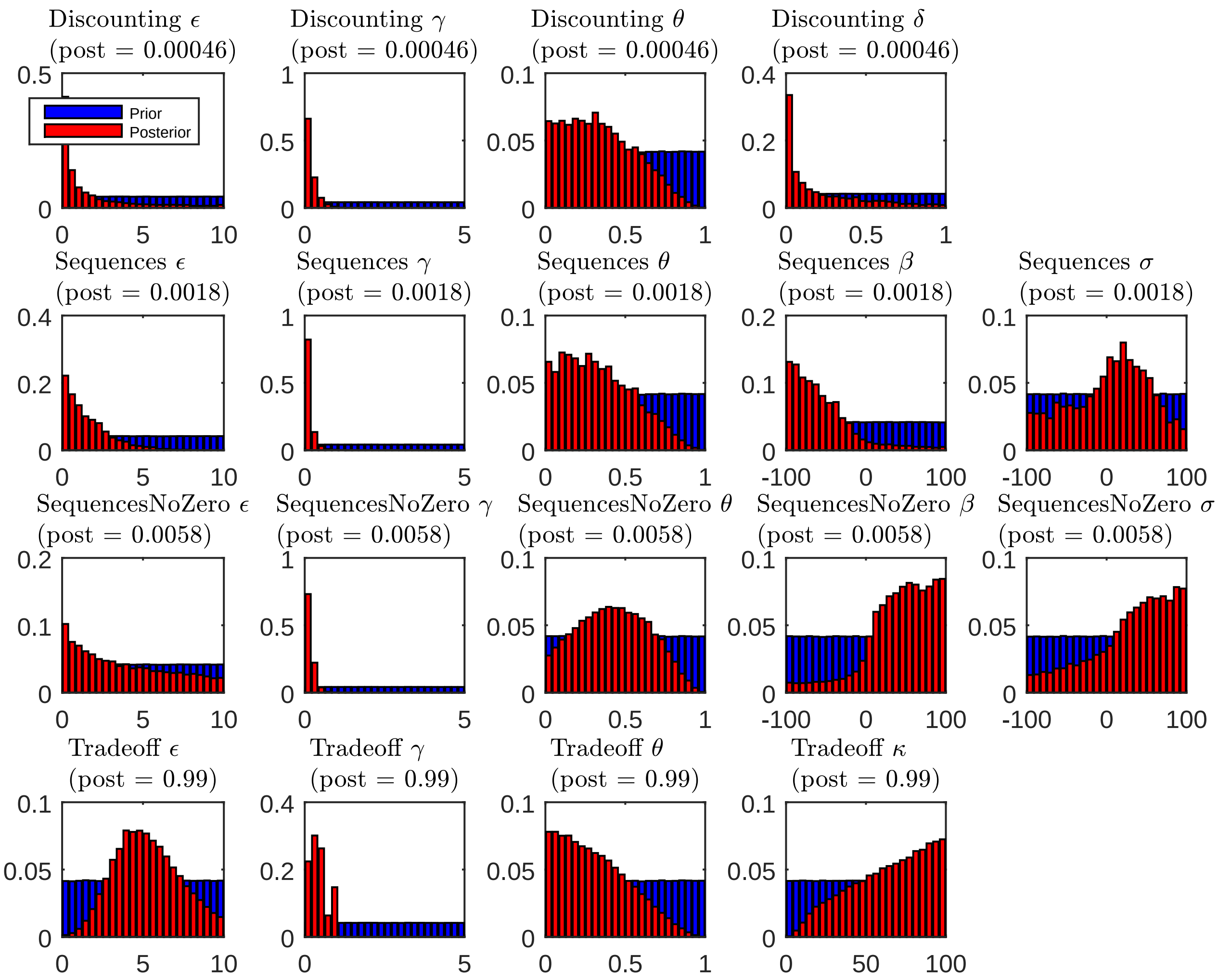
Discounting $\epsilon$
(post = 0.00046)
Prior
Posterior
Discounting $\gamma$
(post = 0.00046)
Discounting $\theta$
(post = 0.00046)
Discounting $\delta$
(post = 0.00046)
Sequences $\epsilon$
(post = 0.0018)
Sequences $\gamma$
(post = 0.0018)
Sequences $\theta$
(post = 0.0018)
Sequences $\beta$
(post = 0.0018)
Sequences $\sigma$
(post = 0.0018)
SequencesNoZero $\epsilon$
(post = 0.0058)
SequencesNoZero $\gamma$
(post = 0.0058)
SequencesNoZero $\theta$
(post = 0.0058)
SequencesNoZero $\beta$
(post = 0.0058)
SequencesNoZero $\sigma$
(post = 0.0058)
Tradeoff $\epsilon$
(post = 0.99)
Tradeoff $\gamma$
(post = 0.99)
Tradeoff $\theta$
(post = 0.99)
Tradeoff $\kappa$
(post = 0.99)
